# Competition between global warming and an abrupt collapse of the AMOC in Earth’s energy imbalance

**DOI:** 10.1038/srep14877

**Published:** 2015-10-06

**Authors:** Sybren Drijfhout

**Affiliations:** 1 Royal Netherlands Meteorological Institute, PO Box 201, 3730AE De Bilt, Netherlands; 2 University of Southampton, National Oceanography Centre Southampton, Waterfront Campus, European Way, Southampton SO14 3ZH, UK

## Abstract

A collapse of the Atlantic Meridional Overturning Circulation (AMOC) leads to global cooling through fast feedbacks that selectively amplify the response in the Northern Hemisphere (NH). How such cooling competes with global warming has long been a topic for speculation, but was never addressed using a climate model. Here it is shown that global cooling due to a collapsing AMOC obliterates global warming for a period of 15–20 years. Thereafter, the global mean temperature trend is reversed and becomes similar to a simulation without an AMOC collapse. The resulting surface warming hiatus lasts for 40–50 years. Global warming and AMOC-induced NH cooling are governed by similar feedbacks, giving rise to a global net radiative imbalance of similar sign, although the former is associated with surface warming, the latter with cooling. Their footprints in outgoing longwave and absorbed shortwave radiation are very distinct, making attribution possible.

Long-term climate change is characterised by a forced response, which can be diagnosed using a two-layer box model^1^,^2^. It is assumed that response and forcing are not in equilibrium, giving rise to a downward energy flux at the top of atmosphere (TOA), and into the ocean in case of positive radiative forcing. The disequilibrium between forcing and response is equal to the amount of heat buried into the deep ocean. Associated with this disequilibrium is a slow component of the response, equivalent to the committed warming after the forcing would return to zero^3^. On decadal timescales unforced variations in ocean heat uptake and TOA energy flux become important as well, with response and feedbacks depending on the ocean state, for instance mixed-layer depth and efficiency of vertical heat exchange between upper and deep ocean^4^, but also the sea surface temperatures (SSTs) in the tropics^5^. On decadal timescales the TOA energy imbalance and ocean heat uptake are very similar, because the heat capacity in other components of the Earth system is small^6^.

When heat transfer to the deep ocean temporarily increases, the response to further increases in radiative forcing can become weak or even zero, giving rise to a climate hiatus. Also a temporal decrease in net radiative forcing might cause a climate hiatus, but on longer timescales one would expect it to be associated with a decrease in downward TOA energy flux and ocean heat uptake instead of an increase, with vertical heat exchange between deep and upper ocean counteracting the radiative forcing. Whether this also holds for shorter (up to decadal) timescales is still unclear, but the signal of a volcanic eruption is reflected in a short-time upward TOA energy pulse in net TOA radiation timeseries^7^.

With respect to the recent climate hiatus (in fact a surface warming hiatus) there is still a strong debate on what is its main cause^6^,^8^,^9^, although there is much evidence for increased ocean heat uptake during the hiatus period^10^,^11^. One of the main drivers for unforced changes in ocean heat uptake is the AMOC. Over large areas in the ocean net heat uptake occurs, which is transferred downward mainly by diffusive processes. To maintain a vertical temperature gradient this heat must be released from the deep to the upper ocean, which occurs in narrow regions of convective mixing^12^. The strength of the heat pump is largely set by the AMOC, determining the ocean’s heat uptake efficiency during transient climate change^13^. On shorter timescales the equilibrium between diffusive downward and convective upward transfer of heat does not hold. Then, a sudden weakening of the AMOC causes reduced convective upward heat transfer, while the diffusive downward transport remains almost unaffected, resulting in net ocean heat uptake^14^.

The heat pump associated with the AMOC also includes surface heat gain over the Southern Hemisphere (SH) upwelling regions^15^,^16^. As a result, wind-driven changes in upwelling also play a role in the AMOC heat pump. For the recent climate hiatus, changes in wind-driven upwelling forced by the trend in Southern Annular Mode appear relevant^17^. A new diagnostic model^18^ based on these processes confirms that a collapse of SH winds collapses the adiabatic pole-to-pole AMOC^19^, leaving a purely diffusive-driven AMOC that is largely confined to the NH, while an abrupt collapse of the pole-to-pole density gradient and a shut-down of convective mixing in the North Atlantic collapses the AMOC completely. The latter process, which is most efficient in causing fast, abrupt changes in AMOC transport is considered here.

For attribution purposes it is necessary that internal variations in ocean heat uptake have a different footprint from the forced response(s). For this reason it is important to study the spatial pattern of these footprints. Separation of the footprints is most easy when the signal associated with unforced variations is large and occurs on shorter timescales than those associated with global warming. An AMOC shutdown serves as an example of how ocean dynamics affect Global Mean Temperature (GMT) and ocean heat uptake. It is an extreme case in which the impact of ocean dynamics on heat uptake efficiency is maximised, noting that increased heat uptake efficiency is linked to the recent climate hiatus^20^.

A shutdown of the AMOC has large climate impacts, with pronounced cooling over the North Atlantic and smaller amounts of warming in the SH^21^,^22^. Although the AMOC only redistributes heat and the climate forcing associated with an AMOC shutdown is zero for the globe as a whole, being anti-symmetric for the two hemispheres, fast climate feedbacks amplify the cooling signal in the NH to a much larger extent than they enhance the warming in the SH^23^,^24^.

One of the characteristics of the AMOC shutdown is a TOA radiation anomaly associated with increased ocean heat uptake^24^,^25^,^26^. In coupled climate models a shutdown of the AMOC is artificially forced by releasing large amounts of freshwater in the North Atlantic^27^, motivated by the fact that climate models cannot yet correctly simulate past abrupt climate change including an AMOC collapse^28^, affecting the likelihood of simulating future abrupt climate change^29^,^30^. None of the previous freshwater hosing experiments investigated the competition between a complete, permanent AMOC collapse, and greenhouse gases forcing acting simultaneously. In one case the AMOC was collapsed during a transient greenhouse scenario run by applying a sudden large freshwater pulse over just one time-step^31^, after which the AMOC recovered from its collapse, leading to a different interplay between radiative forcing and ocean dynamics from what is discussed here. Here, the impact of an AMOC collapse is analysed from an experiment in which persistent hosing, forcing the AMOC to remain in a collapsed state, is combined with increasing CO_2_ forcing, focusing on how they modify the ocean heat uptake and TOA radiation imbalances, and how these anomalies translate into different surface warming signals.

## Results

### Experimental set-up

Data is taken from a five-member ensemble of simulations performed with the coupled climate model ECHAM5/MPI-OM^32^,^33^. The model ensemble uses historical forcing of greenhouse gases and aerosols between years 1950 and 2000. After year 200 it follows the SRES A1B scenario, with each member starting in 1950 with slightly different initial conditions. On January 1, 2000, an AMOC collapse is enforced by adding permanently a freshwater anomaly of 1 Sv (Sverdrup = 10^6^ m^3^ s^−1^) between 50°N and 70°N in the Atlantic. All simulations end in 2100, hundred years after the onset of the AMOC collapse. Also, a control ensemble without freshwater forcing following the same SRES A1B scenario has been carried out and this ensemble has been used for comparison.

### Heat uptake and surface temperature response

During the first fifteen years the greenhouse gas emissions from the SRES A1B scenario only slightly modify the response to the AMOC collapse, which dominates temperature and radiation changes. This response, without modification due to increased greenhouse gases, has been discussed before in other models^26^,^27^,^34^,^35^ and also using the present set of model runs analysed here^22^,^23^,^24^,^36^.

**Figure 1:**
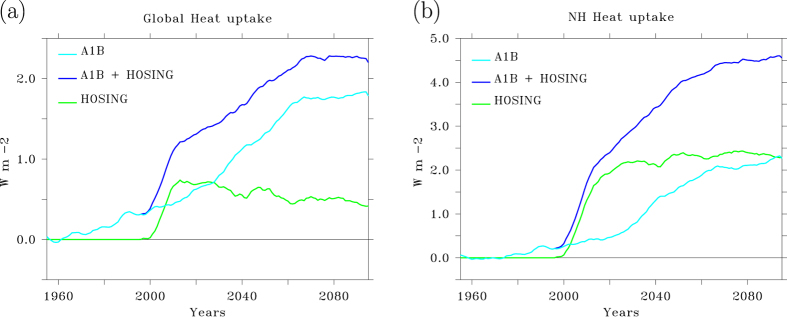
The heat uptake response. (**a**) Time series of global mean heat uptake anomaly relative to the 1950–1960 average, for the A1B scenario run without (A1B) and with additional freshwater hosing (A1B+hosing), and the heat uptake anomaly associated with the shutdown of the AMOC (hosing), all smoothed with an 11-year Welch filter. (**b**) Same, but for the Northern Hemispheric mean. This figure has been created with the free Ferret software package developed by the National Oceanic and Atmospheric Administration (NOAA) and available from www.ferret.noaa.gov.

In the fifty years before the AMOC collapse (1950–2000), a TOA radiation imbalance develops with outgoing radiation being smaller than incoming radiation. The TOA radiation imbalance goes hand in hand with net ocean heat uptake^6^. In response to an AMOC collapse, net ocean heat uptake, that was already keeping the planet’s surface cooler than its equilibrium value associated with the increasing radiative forcing, is further enhanced (Fig. 1a).

In response to the AMOC collapse heat transport across the equator decreases with 0.6 10^15^ W within 15 years. For the NH this decrease in heat transport is equivalent to a drop in energy transport of about 0.15 Wm^−2^ per year, implying a much stronger trend than that associated with the anthropogenic greenhouse forcing, where a doubling of CO_2_ concentrations is equivalent to a radiative forcing of 3.7 Wm^−2^, implying a trend of at most 0.04 Wm^−2^ per year, even less in the first half of the 21^st^ century. Although ocean heat convergence and radiative forcing are not completely comparable, these numbers give a correct indication of magnitudes and timescales involved. The drop in surface forcing of 2 Wm^−2^ in 15 years, associated with the decreased northward heat transport from the SH to the NH translates into a fast increase in ocean heat uptake of 2 Wm^−2^ when the NH is considered in isolation (Fig. 1b). The globally averaged increase in heat uptake is 0.7 Wm^−2^, indicating ocean heat loss in the SH. These changes are about 3 (global) to 10 (NH) times larger than the increase in radiatively forced increase in heat uptake over the same period.

**Figure 2:**
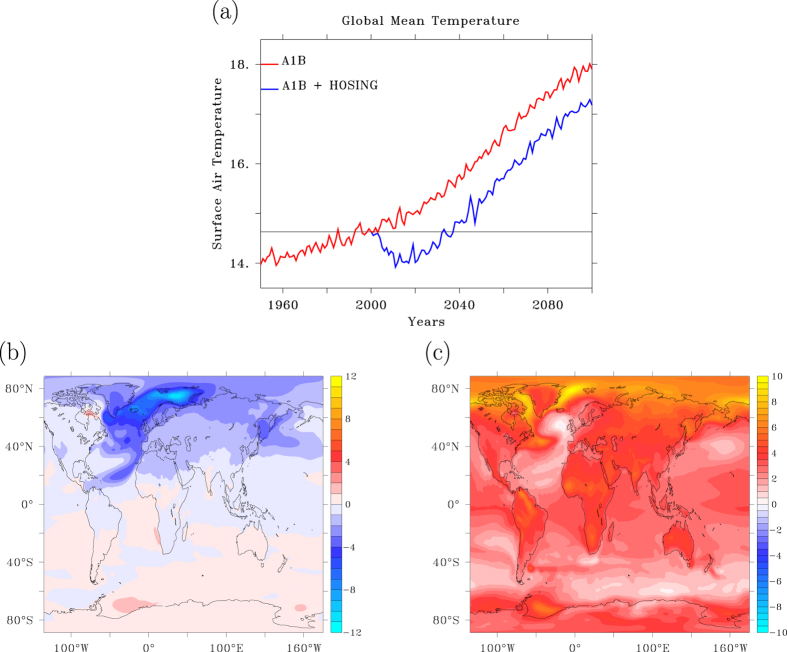
Temperature response to hosing and radiative forcing. (**a**) Annual mean time series of global mean temperature in °C in the A1B climate change scenario with and without an AMOC collapse. (**b**) Temperature anomaly after 15 years since the onset of the AMOC collapse, smoothed with an 11-year Welch filter. (**c**) Same as (**b**) but for the temperature difference between years 95 and 15, showing the footprint of the response to radiative forcing. This figure has been created with the free Ferret software package developed by NOAA.

The increase in greenhouse gases is too slow to compete with the response of an abrupt shutdown of the AMOC, which is reflected in the temperature response of the planet as a whole, although reduced ocean heat transport does not impose a net forcing on the globe. Figure 2a shows the evolution of GMT under an SRES A1B scenario, with and without the forced abrupt collapse of the AMOC. In the latter case, GMT immediately starts to decrease, reaching a minimum after 11 years that is 0.7 °C cooler compared to the year when the AMOC collapse started. When short fluctuations are filtered out, the minimum occurs somewhat later, after 15 years, and mounts to 0.6 °C cooling. Without the forced AMOC collapse GMT increases by 0.3 °C after 15 years, indicating that the negative trend in radiative forcing associated with an AMOC collapse is roughly three times stronger than the upward trend due to increased greenhouse gases. After 15 years the negative temperature trend halts, and after 20 years it reverses and becomes comparable with the trend observed in the SRES A1B without an AMOC shutdown, but with an almost constant offset.

The length of the modelled hiatus period is governed by the time for increased greenhouse forcing to overcome a negative forcing of 0.7 Wm^−2^. For the SRES A1B scenario this period is roughly 40 years. If the AMOC collapse would develop slower (in the order of 40–50 years), GMT would have been characterised with a period of 40 years of zero temperature rise, after which global warming had resumed. If the AMOC collapse would have developed even slower, the increase in heat uptake would not have been able to compensate the increased radiative forcing and GMT would have been characterised with a positive trend, albeit weaker than in case of no AMOC shutdown. It should be noted that other ocean circulation changes may also affect net ocean heat uptake^17^,^37^,^38^.

### Footprints in temperature. a) AMOC collapse

Both an AMOC collapse and radiative forcing are associated with net ocean heat uptake, but the first features global cooling, while the second features global warming. As a result, their spatial temperature patterns are very different (Fig. 2b,c). Apart from the sign difference, the temperature footprint linked to an AMOC collapse shows much more small-scale structure and is asymmetric across the equator (Fig. 2b). A very similar pattern (0–10 year average after the AMOC collapse) was found in Fig. 4 of ref. 31, although due to differences in experimental set-up the responses are not fully comparable.

In response to reduced convective mixing in and over the Labrador Sea, SST and surface air temperature (SAT) anomalies develop which are immediately coupled to each other via air-sea heat exchange. These anomalies are spread eastward by prevailing winds and southward by ocean currents that circulate anti-clockwise in the subpolar gyre. After about 5 years a new minimum develops over the Greenland Sea, north of Iceland, where a second region of deep-water formation is affected. The declining inflow of the North Atlantic Drift into the Arctic induces a tongue of cold SST and SAT spreading northward east of Svalbard. After about 10 years ice-albedo feedbacks cause a temperature minimum east of Svalbard that will persist for a century. Also, a tongue of cold air progressing south-westwards from the North African coast can be seen. Through a wind-evaporation-SST (WES)-feedback^39^ enhanced trade winds cool the surface of the ocean, which on its turn enhances the trades. During this process the Intertropical Convergence Zone (ITCZ) shifts southward^21^,^22^,^33^,^40^ with in its wake a tongue of colder and drier air. A similar scenario may develop spontaneously, i.e. without freshwater hosing, when ocean-sea ice-atmospheric blocking feedbacks are strong enough to collapse Labrador Sea convection^41^.

### b) Radiative forcing

The temperature footprint associated with the radiative response (Fig. 2c) is of larger scale and does not deviate significantly from the pattern associated with global warming without an induced AMOC collapse. Interestingly a small warming hole^42^ in the North Atlantic subpolar gyre is still present, suggesting that a slower adjustment of the North Atlantic circulation takes place after the AMOC abruptly collapsed.

The AMOC temperature-footprint implies that the hemispheric temperature difference reverses sign, with the SH becoming 1 °C warmer than the NH, instead of being more than 1 °C colder, supporting a previous conclusion that this temperature difference is almost entirely due to the AMOC^43^. Immediately after the collapse has been completed, however, the NH increases its temperature faster than the SH in response to anthropogenic warming, as evidenced by the radiative forcing-footprint and 40 years later the NH already has become warmer again than the SH. This response underscores that land-sea contrasts play an important role too in establishing the hemispheric temperature difference, and that the equilibrium hemispheric temperature difference cannot be assessed from transient runs where the climate is not in equilibrium with the forcing, as it involves ocean heat uptake and radiative adjustment on a millennium time scale.

### Footprints in heat uptake. a) AMOC collapse

**Figure 3:**
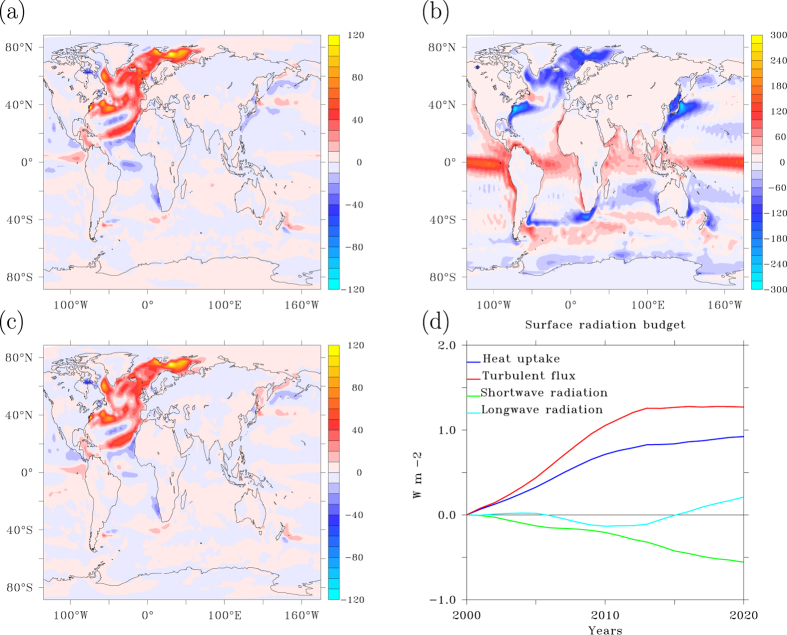
The heat uptake response after an AMOC collapse. (**a**) Heat uptake anomaly in W m^−2^ after 15 years since the onset of the AMOC collapse, smoothed with an 11-year Welch filter. (**b**) Heat uptake climatology for years 1950–2000. (**c**) Same as a) for the anomalous turbulent heat flux. Positive values are into the ocean. (**d**) Global mean heat uptake anomaly and its separate contributions relative to year 2000 for the A1B scenario with additional freshwater hosing, smoothed with an 11-year Welch filter. This figure has been created with the free Ferret software package developed by NOAA.

The spatial patterns of heat uptake and SAT anomaly after 15 years from the onset of the AMOC shutdown are very similar (Figs 2b and 3a), almost each others mirror image. The temperature response is associated with the collapse of Labrador Sea and Greenland Sea convection. The added freshwater reduces the density of surface waters, preventing convective instability. Increasingly smaller amounts of relatively warm water are mixed upward by deep ocean convection and the associated convective heat loss to the overlying atmosphere wanes. This process is illustrated by comparing the heat uptake anomaly and climatology (Fig. 3a,b), clearly showing that north of 25°N increased ocean heat uptake occurs over regions of annual mean heat loss to the atmosphere. This implies that net ocean heat uptake due to an AMOC reduction is predominantly reduced ocean heat loss to the atmosphere. The temperature different between upper and deep ocean is maintained by a balance between downward heat transport by diffusive mixing and upward heat transport associated with convective overturning. It is the latter process that dominates the change in net ocean heat uptake, also in climate change simulations in which the AMOC does not collapse^13^.

Despite the strong similarities between heat uptake and surface temperature, changes in longwave radiation hardly affect the pattern of heat uptake associated with an AMOC collapse. Also changes in net shortwave radiation at the surface cannot compete with the changed turbulent heat fluxes (Fig. 3d). The total heat uptake pattern is completely dominated by reduced turbulent heat loss (Fig. 3c), mostly accomplished by the latent heat associated with reduced evaporation. Increased sea-ice cover implies colder and drier air with less evaporation. As a result, the deep ocean heats up and the atmosphere and surface ocean are cooled.

### b) Radiative forcing

**Figure 4:**
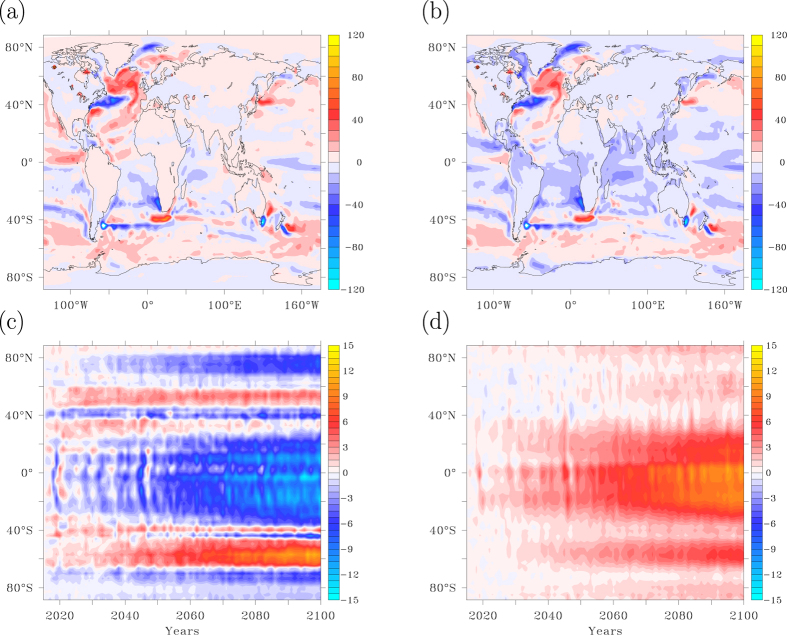
Heat uptake response to radiative forcing. (**a**) Heat uptake anomaly in W m^−2^ between 95 and 15 years after the onset of the AMOC collapse, smoothed with an 11-year Welch filter, illustrating the imprint of radiative forcing versus Fig. 3 where the imprint of the AMOC collapse is shown. (**b**) Same as (**a**), but for the turbulent heat fluxes. (**c**) Zonally averaged turbulent heat flux anomaly relative to year 2015 as a function of time. (**d**) Same as (**c**) for longwave radiation. This figure has been created with the free Ferret software package developed by NOAA.

Global warming reinforces the heat uptake anomaly and the globally averaged heat uptake anomaly increases further after the AMOC collapse is completed (15 years). The small scales of this pattern are still dominated by reduced turbulent heat loss, especially over the North Atlantic subpolar gyre, and associated with further ocean adjustment to the collapsing AMOC (Fig. 4a,b). Globally integrated, however, turbulent heat loss increases after 15 years due to global warming, counteracting the increase in ocean heat uptake. This occurs especially at low and polar latitudes, while in mid latitudes and subpolar regions the turbulent heat loss continues to decrease. When the fast feedbacks associated with the collapse of the AMOC no longer prevail over the slower response to enhanced greenhouse gas concentrations, changes in longwave radiation become the dominant factor in enhancing ocean heat uptake (Fig. 4c,d). As can be seen from Fig. 4d this especially hold for the tropics, where the oceans take up most of the increase in back radiation due to enhanced greenhouse gas concentrations.

### The response at TOA. a) AMOC collapse

**Figure 5:**
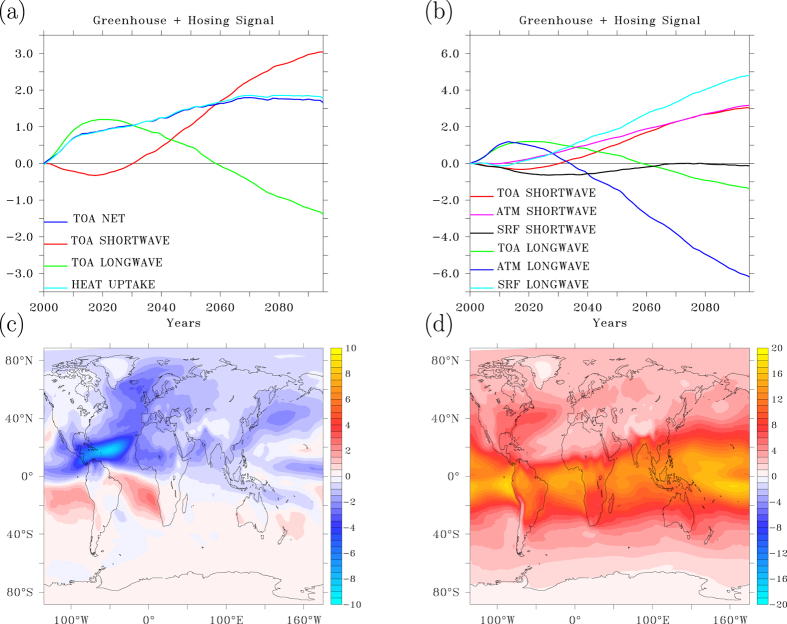
The greenhouse effect. (**a**) Top of atmosphere global mean radiation anomalies in W m^−2^ relative to year 2000, smoothed with an 11-year Welch filter. (**b**) Same as a) but decomposed into surface radiation anomalies, anomalous radiation emitted by the troposphere and top of atmosphere radiation anomalies. (**c**) Water vapour content anomaly in kg m^−2^ after 15 years from the onset of an AMOC collapse, smoothed with an 11-year Welch filter. (**d**) Same as (**c**) but for the difference between years 95 and year 15. This figure has been created with the free Ferret software package developed by NOAA.

During the AMOC collapse a downward radiation anomaly develops, governed by reduced outgoing longwave radiation (OLR) and only partly cancelled by decreased absorbed solar radiation (ASR) (Fig. 5a,b). At the surface both net upward shortwave and longwave radiation increase, due to higher surface albedo and reduced downward longwave radiation associated with a weaker greenhouse effect, despite increasing CO_2_ concentrations, because the atmosphere initially dries and contains less water vapour in response to the reduced evaporation (Fig. 5c). Although water vapour increases in the SH, it decreases much more in the NH.

### b) Radiative forcing

After 15 years, however, this scenario drastically changes: water vapour increases everywhere in response to the increasing CO_2_ concentrations. Eventually the NH negative water vapour anomaly resulting from an AMOC collapse is obliterated (Fig. 5d). When the response to increased CO_2_ overrules the fast atmospheric feedback to an AMOC collapse, enhanced longwave back radiation and decreasing surface albedo cause ASR and OLR to increase at TOA. They counteract the trends induced by an AMOC collapse, but in such a way that the net downward radiation anomaly keeps increasing (Fig. 5a). Most interesting is that the net downward TOA anomaly associated with ocean-induced surface cooling and the one associated with increased radiative forcing have such different characteristics. In case of cooling due to changed dynamics (which occurs by internal variability) the net downward TOA flux is accomplished by reduced OLR, partially counteracted by reduced absorbed shortwave radiation (ASR). In case of increased radiative forcing the net downward TOA flux is accomplished by larger ASR, partially counteracted by increased OLR^44^. This characteristic also features the CMIP5 ensemble-mean response to historical and RCP forcing as can be evidenced with the use of the KNMI climate explorer^45^.

### Footprints in OLR and ASR

**Figure 6:**
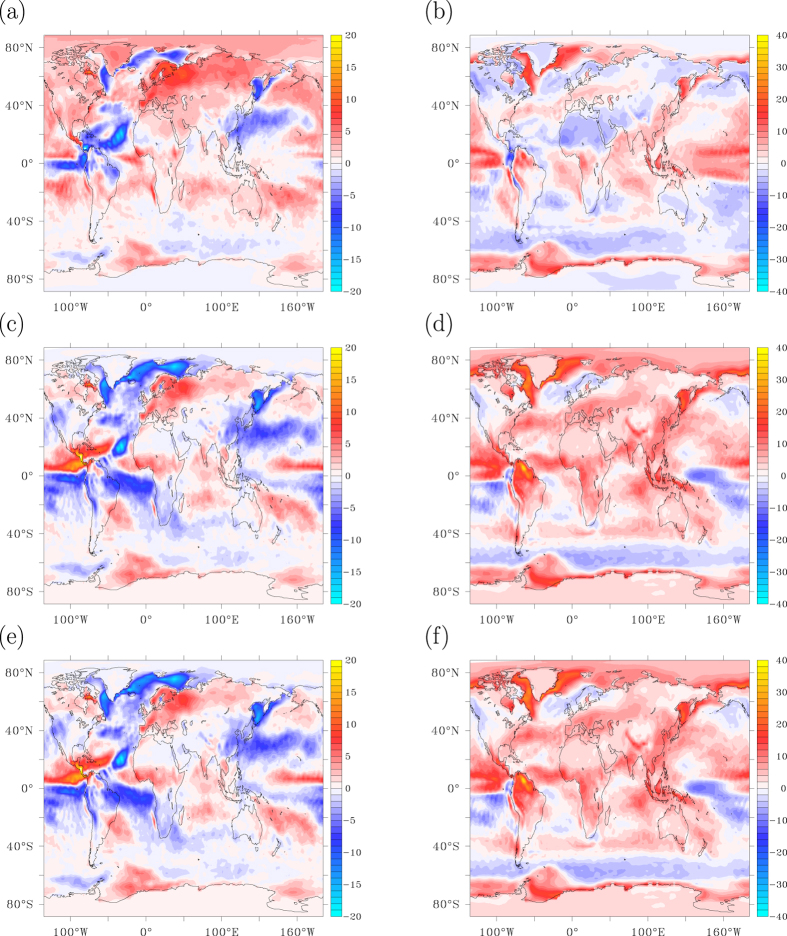
TOA footprints of AMOC collapse and radiative forcing. (**a**) Downward TOA radiation in W m^−2^ anomaly after 15 years, smoothed with an 11-year Welch filter: the footprint of the AMOC collapse. (**b**) Same as (**a**) for the anomaly between years 95 and 15: the footprint of radiative forcing. (**c**) Same as (**a**) for ASR. (**d**) Same as (**b**) for ASR. (**e**) Same as (**a**) for OLR. (**f**) Same as (**b**) for OLR. This figure has been created with the free Ferret software package developed by NOAA.

Figure 6 shows the spatial pattern of the net TOA downward radiation anomaly, OLR and ASR for the AMOC shutdown (Fig. 6a,c,e) and the radiative response (Fig. 6b,d,f). A comparison of the patterns of Fig. 6 with observations of recent TOA radiation anomalies^7^ does not lead to attribution of the ***recent*** climate hiatus to one single cause. Enhanced radiative forcing dominates the pattern of the downward TOA anomaly, partly associated with the recovery from the Pinatubo eruption. There are also signals, especially the dipole over the South Atlantic in ASR and OLR that are consistent with a reduced AMOC. Many aspects of the observed anomalies, however, cannot be related to each of the patterns shown in Fig. 6, indicating multiple drivers, possibly both in radiative forcing^8^,^9^ as well in ocean-atmosphere dynamics^17^,^37^,^38^,^46^,^47^. The globally averaged time series of TOA radiation^48^, however, indicates a role for unforced ocean heat-uptake changes. Firstly, the observed increased TOA downward flux, when being a response to radiative forcing only, would have been associated with a surge in global mean temperature instead of a hiatus. In addition there are two periods where the change in OLR clearly deviates from counteracting changes in ASR, namely 1997–2003 and from 2007 onwards (Fig. 4 from ref. 48).Changes in TOA radiative energy flux correlate with changes in ocean heat content, but are weaker and reflect a smoother and damped image of the implied ocean heat uptake^48^ and estimates from an ocean reanalysis^10^. The periods where OLR deviates from the expected response to radiative forcing indeed coincide with periods where ocean heat-content strongly increases^48^. For an unequivocal attribution, similar patterns as shown in Fig. 6 have to be calculated for different ocean/atmosphere processes that give rise to ocean heat uptake changes, such as changes in ENSO^49^. Also, the response patterns to volcanic eruptions and pattern changes in aerosol emissions would have to be assessed. In addition, for weaker internal variations in heat uptake the relation between TOA anomalies and anomalous upper ocean heat content change was found to consist of OLR and ASR anomalies of similar sign, with OLR dominating but lagging ASR^14^. This underscores the fact that the observed changes in TOA probably consist of a combination of processes with each its own footprint and that we need to establish many more of these footprints for attribution purposes when many processes are acting in concert.

## Discussion

The cooling resulting from an AMOC collapse can locally counteract for more than a century the global warming associated with increased greenhouse gas concentrations. The globally averaged cooling signal, however, is limited in time to several decades and GMT trends eventually reverse sign due to the greenhouse gas forcing. This scenario will apply to almost every other (external) forcing that is able to temporarily evoke global cooling, for instance reduced solar forcing or volcanic eruptions. In case of an AMOC shutdown global cooling sets in when the sum of net radiative forcing and ocean heat transport convergence in the NH display a negative trend. However, the time over which the radiative forcing can decrease in the NH appears to be limited to at most a few decades as long as the greenhouse gas forcing increases with its present-day rate.

Most aspects of this response must be considered model robust. Patterns of cooling associated with a complete AMOC collapse are similar in fully coupled climate models^21^,^24^,^26^,^40^, while the response to a prescribed smaller amount (0.1 Sv) of freshwater hosing is very model-dependent because the modelled AMOC response shows a large spread^27^. The qualitative response to a forced AMOC shut-down will depend on how well the fast atmospheric and coupled ocean-atmosphere feedbacks are represented, while the quantitative response depends on the strength of the AMOC before the collapse and its northward associated heat transport. In the present model ensemble the AMOC is somewhat stronger than observed^50^ (22 Sv versus 19 Sv), although the latter estimate will be less if one includes the period after 2008 when the AMOC sharply declines. On the other hand the cross-equatorial heat transport associated with the AMOC in the model is 0.6 PW, which is lower than estimated from observations as coarse-resolution ocean models display overly diffusive thermoclines, underestimating the vertical heat contrast^51^. As a result, the quantitative response to an AMOC collapse consists of compensating biases. This response (without including the effect of greenhouse gas forcing) is further discussed in ref. 24. It should also be noted that a recent estimate of twenty-century AMOC trends^52^ suggests a stronger AMOC before the RAPID-MOCHA monitoring array^50^ was established, implying a much more pronounced slowdown of the AMOC than occurring in model simulations of the twentieth century.

**Figure 7:**
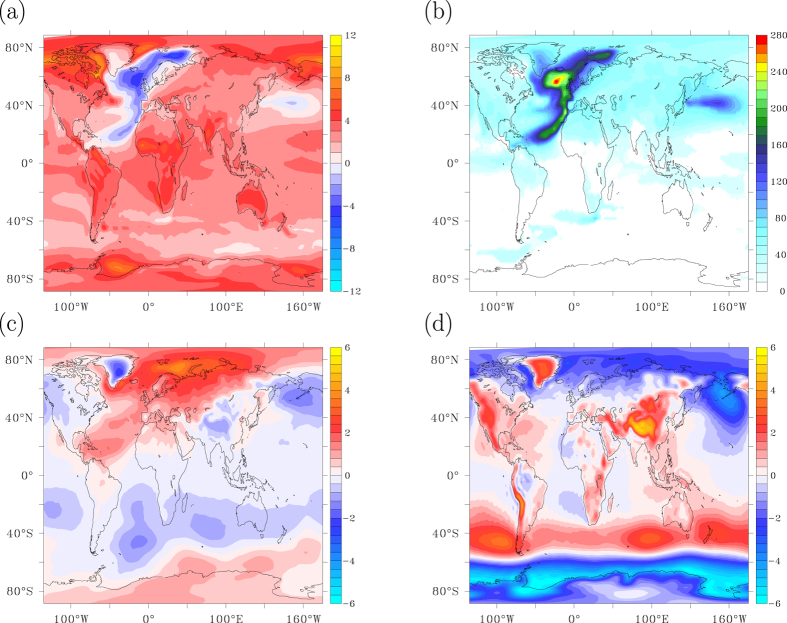
The combined response: dynamical adjustments. (**a**) Temperature anomaly after 95 years from the onset of an AMOC collapse, smoothed with an 11-year Welch filter. (**b**) Recovery time, defined as the time needed for surface air temperature to recover its values from 1990–2000. (**c**) Sea level pressure response in hPa after 15 years from the onset of an AMOC collapse, smoothed with an 11-year Welch filter. (**d**) Same as (**c**) for the anomaly between years 95 and 15. This figure has been created with the free Ferret software package developed by NOAA.

In case of an AMOC collapse, ninety-five years after its onset the 10-year averaged temperature anomaly has become positive in most areas due to increasing radiative forcing, but there are still areas over the North Atlantic and North Pacific where the temperature anomaly is still negative, despite the 95-year long increase in atmospheric CO_2_ concentrations (Fig. 7a). Remarkable is the concentration of a negative SAT anomaly in the eastern North Atlantic, a feature not yet visible after 15 years (compare Figs. 2b and 7a). Associated with the cold tongue in surface temperatures below the enhanced trade winds, a pressure anomaly develops with north-easterly winds at the eastern side of the basin, and predominantly southerly winds and the western side (Fig. 7b). This atmospheric circulation pattern acts to concentrate the cold air anomaly over the North Atlantic along its eastern side. Although the atmospheric circulation anomaly has already developed after 15 years, the temperature adjustment to the wind anomaly has not yet matured after 15 years and only fully develops in the decades following the AMOC collapse, see also Fig. 2c.

Another conspicuous feature is the negative temperature over the subpolar Pacific. Already after 15 years a standing n = 1 wave pattern arises in the NH, associated with a thermal high-pressure cell over the North Atlantic (Fig. 7c). This wave pattern is characterised by low pressures over the North Pacific, evoking enhanced upwelling of colder subsurface water there. The atmospheric wave pattern and the associated Ekman response in the ocean maintain cold SST and SAT over the central North Pacific for many decades. During the years that follow the AMOC collapse, the high pressures over the North Atlantic and Arctic slowly erode, but the low pressures over the Pacific are enhanced (Fig. 7d). After 95 years the pressure response is dominated by lower pressures at high latitudes and higher pressures over the SH mid-latitudes, where a strong enhancement of the westerlies occurs. Interestingly, an area in the SH with retarded warming can be found just over the Agulhas Retroflection (Fig. 7b), associated with a weakening of Agulhas leakage in response to the AMOC collapse^53^. Inspection of the footprints in TOA radiation imbalance shown in Fig. 6b,d,f indeed confirms how these longer-term changes in atmosphere-ocean dynamics affect the response to increasing radiative forcing. Note that these 5-member ensemble mean responses all exceed three standard deviations being significant for a 99% confidence interval.

By extrapolating temperature trends of the last 50 years the recovery time can be estimated, defined as the time needed for SAT to recover its 10-year average from before the AMOC collapse, equivalent to the time a surface climate hiatus due to an AMOC collapse locally persists under an SRES A1B scenario. It is evident from Fig. 7b that it takes several decades for the NH to recover, with areas over the ocean and northwest Europe where recovery takes more than a century. In an elongated band along the eastern North Atlantic a hiatus period may even exceed 200 years. These areas of prolonged cooling are governed by a slow adjustment of the ocean circulation to the collapsed AMOC, enhancing via positive feedbacks the temperature signal which develops during the fast, abrupt AMOC shutdown.

Although the AMOC collapse and anthropogenic radiative forcing have markedly different timescales, this slow ocean adjustment, mediated by a fast atmospheric response to the AMOC collapse and partially amplified by slower ocean-atmosphere feedbacks acting in series, affects the footprint of radiative forcing in ocean heat uptake and TOA energy imbalance, making a complete separation between radiative forcing and internal variations impossible. A much more severe factor hampering attribution of hiatus periods, however, is the large quantitative disagreement between observed TOA radiative fluxes and ocean heat uptake^4^,^39^, suggesting strong biases, instrumental error and large uncertainty bands. To resolve these imperfections more investigation is needed into the spatial patterns of TOA radiative fluxes associated with radiative forcing, feedbacks and internal variations in ocean heat uptake, along with improved analysis of satellite measurements of the Earth’s energy imbalance and better observations of ocean heat content.

## Additional Information

**How to cite this article**: Drijfhout, S. Competition between global warming and an abrupt collapse of the AMOC in Earth's energy imbalance. *Sci. Rep.* **5**, 14877; doi: 10.1038/srep14877 (2015).
